# Supplementary material for: Incidence trends of gastric cancer in the United States over 2000–2020: A population-based analysis
Source: PLoS One. 2024 Sep 25;19(9):e0310040. doi: 10.1371/journal.pone.0310040 (PMC11423999; doi:10.1371/journal.pone.0310040)
Supplement: S1 Table — (DOCX) [file pone.0310040.s001.docx]

**S1 Table.** Results of the tests of parallelism for gastric cancer incidence rate over 2000-2019 in the United States.

| Type | Race | Age | sex | Type | Race | Age | sex | P value |
| --- | --- | --- | --- | --- | --- | --- | --- | --- |
| Cohort 1 | | | | Cohort 2 | | | |  |
| Gastric cancer | NHB | All | Male | Gastric cancer | NHB | <55 | Male | 0.39 |
| Gastric cancer | NHB | All | Male | Gastric cancer | NHB | ≥55 | Male | 0.25 |
| Gastric cancer | NHB | <55 | Male | Gastric cancer | NHB | ≥55 | Male | 0.42 |
| Carcinoid tumor | All | All | Both | Carcinoid tumor | All | <55 | Both | 0.09 |
| Carcinoid tumor | All | All | Female | Carcinoid tumor | All | <55 | Female | 0.0506 |
| Carcinoid tumor | All | All | Female | Carcinoid tumor | All | ≥55 | Female | 0.22 |
| Carcinoid tumor | All | All | Male | Carcinoid tumor | All | <55 | Male | 0.09 |
| Carcinoid tumor | All | All | Male | Carcinoid tumor | All | ≥55 | Male | 0.54 |
| Carcinoid tumor | All | <55 | Male | Carcinoid tumor | All | ≥55 | Male | 0.08 |
| Carcinoid tumor | Hispanic | All | Both | Carcinoid tumor | Hispanic | <55 | Both | 0.57 |
| Carcinoid tumor | Hispanic | All | Both | Carcinoid tumor | Hispanic | ≥55 | Both | 0.06 |
| Carcinoid tumor | Hispanic | <55 | Both | Carcinoid tumor | Hispanic | ≥55 | Both | 0.32 |
| Carcinoid tumor | Hispanic | All | Female | Carcinoid tumor | Hispanic | <55 | Female | 0.63 |
| Carcinoid tumor | Hispanic | All | Female | Carcinoid tumor | Hispanic | ≥55 | Female | 0.26 |
| Carcinoid tumor | Hispanic | <55 | Female | Carcinoid tumor | Hispanic | ≥55 | Female | 0.29 |
| Carcinoid tumor | Hispanic | All | Male | Carcinoid tumor | Hispanic | <55 | Male | 0.09 |
| Carcinoid tumor | Hispanic | All | Male | Carcinoid tumor | Hispanic | ≥55 | Male | 0.53 |
| Carcinoid tumor | Hispanic | <55 | Male | Carcinoid tumor | Hispanic | ≥55 | Male | 0.08 |
| Carcinoid tumor | NHB | All | Both | Carcinoid tumor | NHB | ≥55 | Both | 0.054 |
| Carcinoid tumor | NHB | All | Male | Carcinoid tumor | NHB | <55 | Male | 0.07 |
| Carcinoid tumor | NHB | All | Male | Carcinoid tumor | NHB | ≥55 | Male | 0.55 |
| Carcinoid tumor | NHB | <55 | Male | Carcinoid tumor | NHB | ≥55 | Male | 0.26 |
| Carcinoid tumor | NHW | All | Female | Carcinoid tumor | NHW | <55 | Female | 0.31 |
| Carcinoid tumor | NHW | All | Female | Carcinoid tumor | NHW | ≥55 | Female | 0.07 |
| Carcinoid tumor | NHW | <55 | Female | Carcinoid tumor | NHW | ≥55 | Female | 0.24 |
| GIST | All | All | Female | GIST | All | <55 | Female | 0.39 |
| GIST | All | All | Female | GIST | All | ≥55 | Female | 0.09 |
| GIST | Hispanic | All | Both | GIST | Hispanic | <55 | Both | 0.14 |
| GIST | Hispanic | All | Both | GIST | Hispanic | ≥55 | Both | 0.63 |
| GIST | Hispanic | <55 | Both | GIST | Hispanic | ≥55 | Both | 0.21 |
| GIST | Hispanic | All | Female | GIST | Hispanic | ≥55 | Female | 0.36 |
| GIST | Hispanic | <55 | Female | GIST | Hispanic | ≥55 | Female | 0.13 |
| GIST | Hispanic | All | Male | GIST | Hispanic | <55 | Male | 0.37 |
| GIST | Hispanic | All | Male | GIST | Hispanic | ≥55 | Male | 0.42 |
| GIST | Hispanic | <55 | Male | GIST | Hispanic | ≥55 | Male | 0.53 |
| GIST | NHB | All | Both | GIST | NHB | <55 | Both | 0.45 |
| GIST | NHB | All | Both | GIST | NHB | ≥55 | Both | 0.14 |
| GIST | NHB | <55 | Both | GIST | NHB | ≥55 | Both | 0.055 |
| GIST | NHB | All | Female | GIST | NHB | <55 | Female | 0.67 |
| GIST | NHB | All | Female | GIST | NHB | ≥55 | Female | 0.65 |
| GIST | NHB | <55 | Female | GIST | NHB | ≥55 | Female | 0.42 |
| GIST | NHB | All | Male | GIST | NHB | <55 | Male | 0.83 |
| GIST | NHB | All | Male | GIST | NHB | ≥55 | Male | 0.07 |
| GIST | NHB | <55 | Male | GIST | NHB | ≥55 | Male | 0.12 |
| GIST | NHW | All | Both | GIST | NHW | ≥55 | Both | 0.45 |
| GIST | NHW | All | Female | GIST | NHW | <55 | Female | 0.49 |
| GIST | NHW | All | Female | GIST | NHW | ≥55 | Female | 0.75 |
| GIST | NHW | <55 | Female | GIST | NHW | ≥55 | Female | 0.81 |
| GIST | NHW | All | Male | GIST | NHW | <55 | Male | 0.09 |
| GIST | NHW | All | Male | GIST | NHW | ≥55 | Male | 0.19 |
| GIST | NHW | <55 | Male | GIST | NHW | ≥55 | Male | 0.07 |
| SRCC | NHB | All | Both | SRCC | NHB | <55 | Both | 0.28 |
| SRCC | NHB | All | Both | SRCC | NHB | ≥55 | Both | 0.71 |
| SRCC | NHB | <55 | Both | SRCC | NHB | ≥55 | Both | 0.28 |
| SRCC | NHB | All | Female | SRCC | NHB | <55 | Female | 0.37 |
| SRCC | NHB | All | Female | SRCC | NHB | ≥55 | Female | 0.66 |
| SRCC | NHB | <55 | Female | SRCC | NHB | ≥55 | Female | 0.30 |
| SRCC | NHB | All | Male | SRCC | NHB | <55 | Male | 0.10 |
| SRCC | NHB | All | Male | SRCC | NHB | ≥55 | Male | 0.19 |
| SRCC | NHB | <55 | Male | SRCC | NHB | ≥55 | Male | 0.12 |
| SRCC | NHW | All | Male | SRCC | NHW | ≥55 | Male | 0.52 |
| Gastric cancer | All | <55 | male | Adenocarcinoma | All | <55 | male | 0.06 |
| Gastric cancer | Hispanic | <55 | Both | Adenocarcinoma | Hispanic | <55 | Both | 0.057 |
| Gastric cancer | Hispanic | <55 | Both | Adenocarcinoma | Hispanic | <55 | male | 0.98 |
| Gastric cancer | Hispanic | <55 | male | SRCC | Hispanic | <55 | male | 0.06 |
| Gastric cancer | NHB | <55 | male | adenocarcinoma | NHB | <55 | male | 0.10 |
| Gastric cancer | NHB | <55 | male | Carcinoid tumor | NHB | <55 | male | 0.054 |
| Gastric cancer | NHW | <55 | male | adenocarcinoma | NHW | <55 | male | 0.12 |
| Adenocarcinoma | ALL | <55 | male | SRCC | ALL | <55 | male | 0.09 |
| Adenocarcinoma | Hispanic | All | male | SRCC | Hispanic | All | male | 0.13 |
| Adenocarcinoma | Hispanic | <55 | male | GIST | Hispanic | <55 | male | 0.06 |
| Adenocarcinoma | Hispanic | <55 | male | SRCC | Hispanic | <55 | male | 0.31 |
| Adenocarcinoma | Hispanic | ≥55 | Both | SRCC | Hispanic | ≥55 | Both | 0.056 |
| Adenocarcinoma | NHB | All | Female | SRCC | NHB | All | Female | 0.26 |
| Adenocarcinoma | NHB | <55 | Female | SRCC | NHB | <55 | Female | 0.22 |
| Adenocarcinoma | NHB | ≥55 | Female | SRCC | NHB | ≥55 | Female | 0.08 |
| Adenocarcinoma | NHW | All | Female | SRCC | NHW | All | Female | 0.08 |
| Adenocarcinoma | NHW | <55 | Both | SRCC | NHW | <55 | Both | 0.20 |
| Adenocarcinoma | NHW | <55 | female | SRCC | NHW | <55 | female | 0.84 |
| Adenocarcinoma | NHW | <55 | Male | SRCC | NHW | <55 | Male | 0.19 |
| Carcinoid tumor | All | All | both | GIST | All | All | both | 0.14 |
| Carcinoid tumor | All | <55 | both | GIST | All | <55 | both | 0.06 |
| Carcinoid tumor | All | All | Female | GIST | All | All | Female | 0.37 |
| Carcinoid tumor | All | <55 | Female | GIST | All | <55 | Female | 0.25 |
| Carcinoid tumor | All | ≥55 | Female | GIST | All | ≥55 | Female | 0.13 |
| Carcinoid tumor | Hispanic | All | Female | GIST | Hispanic | All | Female | 0.57 |
| Carcinoid tumor | Hispanic | <55 | Both | GIST | Hispanic | <55 | Both | 0.76 |
| Carcinoid tumor | Hispanic | <55 | Female | GIST | Hispanic | <55 | Female | 0.77 |
| Carcinoid tumor | Hispanic | <55 | Male | GIST | Hispanic | <55 | Male | 0.48 |
| Carcinoid tumor | Hispanic | ≥55 | Both | GIST | Hispanic | ≥55 | Both | 0.051 |
| Carcinoid tumor | Hispanic | ≥55 | Female | GIST | Hispanic | ≥55 | Female | 0.46 |
| Carcinoid tumor | NHB | All | Male | GIST | NHB | All | Male | 0.051 |
| Carcinoid tumor | NHB | <55 | both | GIST | NHB | <55 | both | 0.66 |
| Carcinoid tumor | NHB | <55 | Female | GIST | NHB | <55 | Female | 0.17 |
| Carcinoid tumor | NHB | <55 | Male | GIST | NHB | <55 | Male | 0.13 |
| Carcinoid tumor | NHB | ≥55 | Male | GIST | NHB | ≥55 | Male | 0.054 |
| Carcinoid tumor | NHW | all | Female | GIST | NHW | all | Female | 0.25 |
| Carcinoid tumor | NHW | <55 | Female | GIST | NHW | <55 | Female | 0.62 |
| Carcinoid tumor | NHW | ≥55 | Both | GIST | NHW | ≥55 | Both | 0.06 |
| Carcinoid tumor | NHW | ≥55 | Female | GIST | NHW | ≥55 | Female | 0.36 |
| Gastric cancer | All | All | Both | Gastric cancer | Hispanic | All | Both | 0.31 |
| Gastric cancer | All | All | Both | Gastric cancer | NHW | All | Both | 0.15 |
| Gastric cancer | All | All | Female | Gastric cancer | NHW | All | Female | 0.46 |
| Gastric cancer | All | All | Male | Gastric cancer | Hispanic | All | Male | 0.35 |
| Gastric cancer | All | <55 | Male | Gastric cancer | NHW | <55 | Male | 0.46 |
| Gastric cancer | Hispanic | <55 | Both | Gastric cancer | NHB | <55 | Both | 0.13 |
| Gastric cancer | Hispanic | <55 | Female | Gastric cancer | NHB | <55 | Female | 0.10 |
| Gastric cancer | Hispanic | <55 | Female | Gastric cancer | NHW | <55 | Female | 0.36 |
| Gastric cancer | Hispanic | <55 | Male | Gastric cancer | NHB | <55 | Male | 0.98 |
| Gastric cancer | Hispanic | <55 | Both | Gastric cancer | NHW | <55 | Both | 0.84 |
| Gastric cancer | Hispanic | <55 | Female | Gastric cancer | NHW | <55 | Female | 0.11 |
| Gastric cancer | Hispanic | <55 | Male | Gastric cancer | NHW | <55 | Male | 0.77 |
| Gastric cancer | Hispanic | ≥55 | Both | Gastric cancer | NHB | ≥55 | Both | 0.51 |
| Gastric cancer | Hispanic | ≥55 | Female | Gastric cancer | NHB | ≥55 | Female | 0.45 |
| Gastric cancer | Hispanic | ≥55 | Female | Gastric cancer | NHW | ≥55 | Female | 0.20 |
| Gastric cancer | Hispanic | ≥55 | Male | Gastric cancer | NHW | ≥55 | Male | 0.08 |
| Gastric cancer | NHB | ≥55 | Female | Gastric cancer | NHB | ≥55 | Female | 0.09 |
| Adenocarcinoma | All | All | Both | Adenocarcinoma | NHW | All | Both | 0.07 |
| Adenocarcinoma | All | <55 | Both | Adenocarcinoma | Hispanic | <55 | Both | 0.39 |
| Adenocarcinoma | All | <55 | Both | Adenocarcinoma | NHW | <55 | Both | 0.15 |
| Adenocarcinoma | All | <55 | Female | Adenocarcinoma | Hispanic | <55 | Female | 0.08 |
| Adenocarcinoma | All | <55 | Male | Adenocarcinoma | Hispanic | <55 | Male | 0.89 |
| Adenocarcinoma | All | <55 | Male | Adenocarcinoma | NHW | <55 | Male | 0.79 |
| Adenocarcinoma | Hispanic | All | Female | Adenocarcinoma | NHB | All | Female | 0.055 |
| Adenocarcinoma | Hispanic | All | Female | Adenocarcinoma | NHW | All | Female | 0.51 |
| Adenocarcinoma | Hispanic | <55 | both | Adenocarcinoma | NHW | <55 | both | 0.55 |
| Adenocarcinoma | Hispanic | <55 | Female | Adenocarcinoma | NHW | <55 | Female | 0.08 |
| Adenocarcinoma | Hispanic | <55 | Male | Adenocarcinoma | NHW | <55 | Male | 0.78 |
| Adenocarcinoma | Hispanic | ≥55 | Female | Adenocarcinoma | NHB | ≥55 | Female | 0.12 |
| Adenocarcinoma | Hispanic | ≥55 | Female | Adenocarcinoma | NHW | ≥55 | Female | 0.34 |
| Adenocarcinoma | NHB | <55 | Female | Adenocarcinoma | NHW | <55 | Female | 0.37 |
| Carcinoid tumor | All | All | Female | Carcinoid tumor | Hispanic | <55 | Female | 0.50 |
| Carcinoid tumor | All | All | Female | Carcinoid tumor | NHB | All | Female | 0.18 |
| Carcinoid tumor | All | All | Female | Carcinoid tumor | NHW | All | Female | 0.13 |
| Carcinoid tumor | All | All | Male | Carcinoid tumor | NHW | All | Female | 0.21 |
| Carcinoid tumor | All | <55 | Both | Carcinoid tumor | Hispanic | All | Male | 0.55 |
| Carcinoid tumor | All | <55 | Both | Carcinoid tumor | NHB | <55 | Both | 0.50 |
| Carcinoid tumor | All | <55 | Both | Carcinoid tumor | NHW | <55 | Both | 0.51 |
| Carcinoid tumor | All | <55 | female | Carcinoid tumor | Hispanic | <55 | Both | 0.82 |
| Carcinoid tumor | All | <55 | female | Carcinoid tumor | NHB | <55 | female | 0.46 |
| Carcinoid tumor | All | <55 | female | Carcinoid tumor | NHW | <55 | female | 0.23 |
| Carcinoid tumor | All | <55 | Male | Carcinoid tumor | Hispanic | <55 | female | 0.17 |
| Carcinoid tumor | All | ≥55 | Both | Carcinoid tumor | Hispanic | <55 | Male | 0.38 |
| Carcinoid tumor | All | ≥55 | Both | Carcinoid tumor | NHW | ≥55 | Both | 0.54 |
| Carcinoid tumor | All | ≥55 | female | Carcinoid tumor | Hispanic | ≥55 | Both | 0.33 |
| Carcinoid tumor | All | ≥55 | female | Carcinoid tumor | NHW | ≥55 | female | 0.60 |
| Carcinoid tumor | All | ≥55 | Male | Carcinoid tumor | NHW | ≥55 | female | 0.15 |
| Carcinoid tumor | Hispanic | All | Both | Carcinoid tumor | NHB | All | Both | 0.38 |
| Carcinoid tumor | Hispanic | All | Both | Carcinoid tumor | NHW | All | Both | 0.63 |
| Carcinoid tumor | Hispanic | All | Female | Carcinoid tumor | NHB | All | Female | 0.15 |
| Carcinoid tumor | Hispanic | All | Female | Carcinoid tumor | NHW | All | Female | 0.1 |
| Carcinoid tumor | Hispanic | All | Male | Carcinoid tumor | NHB | All | Male | 0.79 |
| Carcinoid tumor | Hispanic | <55 | Both | Carcinoid tumor | NHB | <55 | Both | 0.97 |
| Carcinoid tumor | Hispanic | <55 | Both | Carcinoid tumor | NHW | <55 | Both | 0.12 |
| Carcinoid tumor | Hispanic | <55 | Female | Carcinoid tumor | NHB | <55 | Female | 0.15 |
| Carcinoid tumor | Hispanic | <55 | Female | Carcinoid tumor | NHW | <55 | Female | 0.63 |
| Carcinoid tumor | Hispanic | <55 | Male | Carcinoid tumor | NHB | <55 | Male | 0.24 |
| Carcinoid tumor | Hispanic | <55 | Male | Carcinoid tumor | NHW | <55 | Male | 0.27 |
| Carcinoid tumor | Hispanic | ≥55 | Both | Carcinoid tumor | NHB | ≥55 | Both | 0.08 |
| Carcinoid tumor | Hispanic | ≥55 | Both | Carcinoid tumor | NHW | ≥55 | Both | 0.13 |
| Carcinoid tumor | Hispanic | ≥55 | Female | Carcinoid tumor | NHW | ≥55 | Female | 0.11 |
| Carcinoid tumor | Hispanic | ≥55 | Male | Carcinoid tumor | NHB | ≥55 | Male | 0.37 |
| Carcinoid tumor | NHB | All | Female | Carcinoid tumor | NHW | All | Female | 0.09 |
| Carcinoid tumor | NHB | <55 | Both | Carcinoid tumor | NHW | <55 | Both | 0.10 |
| Carcinoid tumor | NHB | <55 | Female | Carcinoid tumor | NHW | <55 | Female | 0.14 |
| Carcinoid tumor | NHB | ≥55 | Male | Carcinoid tumor | NHW | ≥55 | Male | 0.09 |
| GIST | All | All | Female | GIST | Hispanic | All | Female | 0.51 |
| GIST | All | All | Female | GIST | NHB | All | Female | 0.35 |
| GIST | All | All | Female | GIST | NHW | All | Female | 0.26 |
| GIST | All | All | Male | GIST | Hispanic | All | Male | 0.32 |
| GIST | All | All | Male | GIST | NHB | All | Male | 0.29 |
| GIST | All | <55 | Both | GIST | Hispanic | <55 | Both | 0.64 |
| GIST | All | <55 | Both | GIST | NHB | <55 | Both | 0.23 |
| GIST | All | <55 | Female | GIST | Hispanic | <55 | Female | 0.58 |
| GIST | All | <55 | Female | GIST | NHB | <55 | Female | 0.53 |
| GIST | All | <55 | Female | GIST | NHW | <55 | Female | 0.05 |
| GIST | All | <55 | Male | GIST | Hispanic | <55 | Male | 0.18 |
| GIST | All | <55 | Male | GIST | NHB | <55 | Male | 0.70 |
| GIST | All | <55 | Male | GIST | NHW | <55 | Male | 0.051 |
| GIST | All | ≥55 | Both | GIST | Hispanic | ≥55 | Both | 0.38 |
| GIST | All | ≥55 | Female | GIST | Hispanic | ≥55 | Female | 0.52 |
| GIST | All | ≥55 | Female | GIST | NHB | ≥55 | Female | 0.30 |
| GIST | All | ≥55 | Female | GIST | NHW | ≥55 | Female | 0.15 |
| GIST | All | ≥55 | Male | GIST | Hispanic | ≥55 | Male | 0.64 |
| GIST | All | ≥55 | Male | GIST | NHB | ≥55 | Male | 0.27 |
| GIST | All | ≥55 | Male | GIST | NHW | ≥55 | Male | 0.13 |
| GIST | Hispanic | All | Both | GIST | NHB | All | Both | 0.75 |
| GIST | Hispanic | All | Female | GIST | NHB | All | Female | 0.93 |
| GIST | Hispanic | All | Female | GIST | NHW | All | Female | 0.35 |
| GIST | Hispanic | All | Male | GIST | NHB | All | Male | 0.77 |
| GIST | Hispanic | <55 | Both | GIST | NHB | <55 | Both | 0.40 |
| GIST | Hispanic | <55 | Both | GIST | NHW | <55 | Both | 0.36 |
| GIST | Hispanic | <55 | Female | GIST | NHB | <55 | Female | 0.75 |
| GIST | Hispanic | <55 | Female | GIST | NHW | <55 | Female | 0.26 |
| GIST | Hispanic | <55 | Male | GIST | NHB | <55 | Male | 0.30 |
| GIST | Hispanic | <55 | Male | GIST | NHW | <55 | Male | 0.50 |
| GIST | Hispanic | ≥55 | Both | GIST | NHB | ≥55 | Both | 0.25 |
| GIST | Hispanic | ≥55 | Both | GIST | NHW | ≥55 | Both | 0.08 |
| GIST | Hispanic | ≥55 | Female | GIST | NHB | ≥55 | Female | 0.94 |
| GIST | Hispanic | ≥55 | Female | GIST | NHW | ≥55 | Female | 0.24 |
| GIST | Hispanic | ≥55 | Male | GIST | NHB | ≥55 | Male | 0.58 |
| GIST | Hispanic | ≥55 | Male | GIST | NHW | ≥55 | Male | 0.49 |
| GIST | NHB | All | Female | GIST | NHW | All | Female | 0.11 |
| GIST | NHB | <55 | Female | GIST | NHW | <55 | Female | 0.50 |
| GIST | NHB | <55 | Male | GIST | NHW | <55 | Male | 0.27 |
| GIST | NHB | ≥55 | Female | GIST | NHW | ≥55 | Female | 0.07 |
| GIST | NHB | ≥55 | Male | GIST | NHW | ≥55 | Male | 0.40 |
| SRCC | All | All | Both | SRCC | Hispanic | All | Both | 0.52 |
| SRCC | All | All | Female | SRCC | Hispanic | All | Female | 0.29 |
| SRCC | All | All | Male | SRCC | Hispanic | All | Male | 0.43 |
| SRCC | All | All | Male | SRCC | NHW | All | Male | 0.0502 |
| SRCC | All | <55 | Both | SRCC | Hispanic | <55 | Both | 0.52 |
| SRCC | All | <55 | Both | SRCC | NHW | <55 | Both | 0.46 |
| SRCC | All | <55 | Female | SRCC | Hispanic | <55 | Female | 0.32 |
| SRCC | All | <55 | Female | SRCC | NHW | <55 | Female | 0.84 |
| SRCC | All | <55 | Male | SRCC | Hispanic | <55 | Male | 0.73 |
| SRCC | All | <55 | Male | SRCC | NHW | <55 | Male | 0.58 |
| SRCC | All | ≥55 | Both | SRCC | Hispanic | ≥55 | Both | 0.75 |
| SRCC | All | ≥55 | Both | SRCC | NHB | ≥55 | Both | 0.74 |
| SRCC | All | ≥55 | Both | SRCC | NHW | ≥55 | Both | 0.33 |
| SRCC | All | ≥55 | Female | SRCC | Hispanic | ≥55 | Female | 0.33 |
| SRCC | All | ≥55 | Female | SRCC | NHB | ≥55 | Female | 0.08 |
| SRCC | All | ≥55 | Male | SRCC | Hispanic | ≥55 | Male | 0.11 |
| SRCC | All | ≥55 | Male | SRCC | NHB | ≥55 | Male | 0.57 |
| SRCC | All | ≥55 | Male | SRCC | NHW | ≥55 | Male | 0.23 |
| SRCC | Hispanic | All | Both | SRCC | NHW | All | Both | 0.90 |
| SRCC | Hispanic | All | Female | SRCC | NHW | All | Female | 0.51 |
| SRCC | Hispanic | All | Male | SRCC | NHB | All | Male | 0.21 |
| SRCC | Hispanic | All | Male | SRCC | NHW | All | Male | 0.62 |
| SRCC | Hispanic | <55 | Both | SRCC | NHW | <55 | Both | 0.98 |
| SRCC | Hispanic | <55 | Female | SRCC | NHW | <55 | Female | 0.32 |
| SRCC | Hispanic | <55 | Male | SRCC | NHW | <55 | Male | 0.92 |
| SRCC | Hispanic | ≥55 | Both | SRCC | NHB | ≥55 | Both | 0.55 |
| SRCC | Hispanic | ≥55 | Both | SRCC | NHW | ≥55 | Both | 0.97 |
| SRCC | Hispanic | ≥55 | Female | SRCC | NHW | ≥55 | Female | 0.35 |
| SRCC | Hispanic | ≥55 | Male | SRCC | NHB | ≥55 | Male | 0.41 |
| SRCC | Hispanic | ≥55 | male | SRCC | NHW | ≥55 | male | 0.20 |
| SRCC | NHB | All | Both | SRCC | NHW | All | Both | 0.18 |
| SRCC | NHB | All | Female | SRCC | NHW | All | Female | 0.45 |
| SRCC | NHB | All | Male | SRCC | NHW | All | Male | 0.74 |
| SRCC | NHB | <55 | Female | SRCC | NHW | <55 | Female | 0.11 |
| SRCC | NHB | ≥55 | Both | SRCC | NHW | ≥55 | Both | 0.93 |
| SRCC | NHB | ≥55 | Female | SRCC | NHW | ≥55 | Female | 0.502 |
| SRCC | NHB | ≥55 | Male | SRCC | NHW | ≥55 | Male | 0.91 |
| Gastric cancer | NHB | ≥55 | Both | Gastric cancer | NHB | ≥55 | Female | 0.72 |
| Gastric cancer | NHB | ≥55 | Both | Gastric cancer | NHB | ≥55 | Male | 0.10 |
| Gastric cancer | NHB | ≥55 | Female | Gastric cancer | NHB | ≥55 | Male | 0.35 |
| Gastric cancer | NHW | ≥55 | Both | Gastric cancer | NHW | ≥55 | Female | 0.12 |
| Adenocarcinoma | All | All | Female | Adenocarcinoma | All | All | Male | 0.06 |
| Adenocarcinoma | Hispanic | All | Both | Adenocarcinoma | Hispanic | All | Female | 0.057 |
| Adenocarcinoma | Hispanic | All | Female | Adenocarcinoma | Hispanic | All | Male | 0.13 |
| Adenocarcinoma | Hispanic | ≥55 | Both | Adenocarcinoma | Hispanic | ≥55 | Female | 0.53 |
| Adenocarcinoma | Hispanic | ≥55 | Both | Adenocarcinoma | Hispanic | ≥55 | Male | 0.65 |
| Adenocarcinoma | Hispanic | ≥55 | Female | Adenocarcinoma | Hispanic | ≥55 | Male | 0.90 |
| Adenocarcinoma | NHB | All | Both | Adenocarcinoma | NHB | All | Female | 0.06 |
| Adenocarcinoma | NHB | All | Both | Adenocarcinoma | NHB | All | Male | 0.08 |
| Adenocarcinoma | NHB | All | Female | Adenocarcinoma | NHB | All | Male | 0.18 |
| Adenocarcinoma | NHB | ≥55 | Both | Adenocarcinoma | NHB | ≥55 | Male | 0.10 |
| Adenocarcinoma | NHW | All | Both | Adenocarcinoma | NHW | All | Male | 0.08 |
| Adenocarcinoma | NHW | <55 | Both | Adenocarcinoma | NHW | <55 | Female | 0.84 |
| Adenocarcinoma | NHW | <55 | Both | Adenocarcinoma | NHW | <55 | Male | 0.37 |
| Adenocarcinoma | NHW | <55 | Female | Adenocarcinoma | NHW | <55 | Male | 0.41 |
| Adenocarcinoma | NHW | ≥55 | Both | Adenocarcinoma | NHW | ≥55 | Male | 0.06 |
| Carcinoid tumor | All | All | Both | Carcinoid tumor | All | All | Female | 0.06 |
| Carcinoid tumor | All | <55 | Both | Carcinoid tumor | All | <55 | Male | 0.12 |
| Carcinoid tumor | All | ≥55 | Both | Carcinoid tumor | All | ≥55 | Male | 0.18 |
| Carcinoid tumor | Hispanic | All | Both | Carcinoid tumor | Hispanic | All | Female | 0.08 |
| Carcinoid tumor | Hispanic | <55 | Both | Carcinoid tumor | Hispanic | <55 | Female | 0.25 |
| Carcinoid tumor | Hispanic | <55 | Both | Carcinoid tumor | Hispanic | <55 | Male | 0.35 |
| Carcinoid tumor | Hispanic | <55 | Female | Carcinoid tumor | Hispanic | <55 | Male | 0.42 |
| Carcinoid tumor | Hispanic | ≥55 | Both | Carcinoid tumor | Hispanic | ≥55 | Female | 0.11 |
| Carcinoid tumor | NHB | All | Both | Carcinoid tumor | NHB | All | Female | 0.30 |
| Carcinoid tumor | NHB | All | Both | Carcinoid tumor | NHB | All | Male | 0.08 |
| Carcinoid tumor | NHB | All | Female | Carcinoid tumor | NHB | All | Male | 0.18 |
| Carcinoid tumor | NHB | ≥55 | Both | Carcinoid tumor | NHB | ≥55 | Female | 0.60 |
| Carcinoid tumor | NHB | ≥55 | Both | Carcinoid tumor | NHB | ≥55 | Male | 0.40 |
| Carcinoid tumor | NHB | ≥55 | Female | Carcinoid tumor | NHB | ≥55 | Male | 0.63 |
| Carcinoid tumor | NHW | All | Both | Carcinoid tumor | NHW | All | Male | 0.08 |
| Carcinoid tumor | NHW | All | Female | Carcinoid tumor | NHW | All | Male | 0.06 |
| Carcinoid tumor | NHW | <55 | Both | Carcinoid tumor | NHW | <55 | Female | 0.13 |
| Carcinoid tumor | NHW | <55 | Female | Carcinoid tumor | NHW | <55 | Male | 0.17 |
| GIST | All | All | Both | GIST | All | All | Female | 0.056 |
| GIST | All | All | Both | GIST | All | All | Male | 0.06 |
| GIST | All | All | Female | GIST | All | All | Male | 0.08 |
| GIST | All | ≥55 | Both | GIST | All | ≥55 | Female | 0.15 |
| GIST | All | ≥55 | Both | GIST | All | ≥55 | Male | 0.16 |
| GIST | All | ≥55 | Female | GIST | All | ≥55 | Male | 0.09 |
| GIST | Hispanic | All | Both | GIST | Hispanic | All | Female | 0.27 |
| GIST | Hispanic | All | Both | GIST | Hispanic | All | Male | 0.49 |
| GIST | Hispanic | All | Female | GIST | Hispanic | All | Male | 0.34 |
| GIST | Hispanic | <55 | Both | GIST | Hispanic | <55 | Female | 0.23 |
| GIST | Hispanic | <55 | Both | GIST | Hispanic | <55 | Male | 0.17 |
| GIST | Hispanic | <55 | Female | GIST | Hispanic | <55 | Male | 0.21 |
| GIST | Hispanic | ≥55 | Both | GIST | Hispanic | ≥55 | Female | 0.84 |
| GIST | Hispanic | ≥55 | Both | GIST | Hispanic | ≥55 | Male | 0.58 |
| GIST | Hispanic | ≥55 | Female | GIST | Hispanic | ≥55 | Male | 0.96 |
| GIST | NHB | All | Both | GIST | NHB | All | Female | 0.78 |
| GIST | NHB | All | Both | GIST | NHB | All | Male | 0.52 |
| GIST | NHB | All | Female | GIST | NHB | All | Male | 0.56 |
| GIST | NHB | <55 | Both | GIST | NHB | <55 | Female | 0.31 |
| GIST | NHB | <55 | Both | GIST | NHB | <55 | Male | 0.59 |
| GIST | NHB | <55 | Female | GIST | NHB | <55 | Male | 0.15 |
| GIST | NHB | ≥55 | Both | GIST | NHB | ≥55 | Female | 0.67 |
| GIST | NHB | ≥55 | Both | GIST | NHB | ≥55 | Male | 0.59 |
| GIST | NHB | ≥55 | Female | GIST | NHB | ≥55 | Male | 0.80 |
| GIST | NHW | All | Both | GIST | NHW | All | Male | 0.20 |
| GIST | NHW | All | Female | GIST | NHW | All | Male | 0.055 |
| GIST | NHW | <55 | Both | GIST | NHW | <55 | Male | 0.55 |
| GIST | NHW | ≥55 | Both | GIST | NHW | ≥55 | Female | 0.43 |
| GIST | NHW | ≥55 | Both | GIST | NHW | ≥55 | Male | 0.42 |
| GIST | NHW | ≥55 | Female | GIST | NHW | ≥55 | Male | 0.40 |
| SRCC | All | <55 | Both | SRCC | All | <55 | Female | 0.07 |
| SRCC | All | ≥55 | Both | SRCC | All | ≥55 | Female | 0.58 |
| SRCC | All | ≥55 | Both | SRCC | All | ≥55 | Male | 0.10 |
| SRCC | All | ≥55 | Female | SRCC | All | ≥55 | Male | 0.06 |
| SRCC | Hispanic | All | Both | SRCC | Hispanic | All | Female | 0.08 |
| SRCC | Hispanic | <55 | Both | SRCC | Hispanic | <55 | Female | 0.38 |
| SRCC | Hispanic | <55 | Both | SRCC | Hispanic | <55 | Male | 0.11 |
| SRCC | Hispanic | <55 | Female | SRCC | Hispanic | <55 | Male | 0.28 |
| SRCC | Hispanic | ≥55 | Both | SRCC | Hispanic | ≥55 | Female | 0.31 |
| SRCC | Hispanic | ≥55 | Both | SRCC | Hispanic | ≥55 | Male | 0.12 |
| SRCC | NHB | All | Both | SRCC | NHB | All | Female | 0.49 |
| SRCC | NHB | All | Both | SRCC | NHB | All | Male | 0.26 |
| SRCC | NHB | All | Female | SRCC | NHB | All | Male | 0.51 |
| SRCC | NHB | <55 | Both | SRCC | NHB | <55 | Female | 0.09 |
| SRCC | NHB | <55 | Both | SRCC | NHB | <55 | Male | 0.20 |
| SRCC | NHB | <55 | Female | SRCC | NHB | <55 | Male | 0.11 |
| SRCC | NHB | ≥55 | Both | SRCC | NHB | ≥55 | Female | 0.75 |
| SRCC | NHB | ≥55 | Both | SRCC | NHB | ≥55 | Male | 0.41 |
| SRCC | NHB | ≥55 | Female | SRCC | NHB | ≥55 | Male | 0.30 |
| SRCC | NHW | All | Both | SRCC | NHW | All | Female | 0.06 |
| SRCC | NHW | All | Both | SRCC | NHW | All | Male | 0.19 |
| SRCC | NHW | All | Female | SRCC | NHW | All | Male | 0.07 |
| SRCC | NHW | <55 | Both | SRCC | NHW | <55 | Male | 0.059 |
| SRCC | NHW | ≥55 | Both | SRCC | NHW | ≥55 | Female | 0.86 |
| SRCC | NHW | ≥55 | Both | SRCC | NHW | ≥55 | Male | 0.71 |
| SRCC | NHW | ≥55 | Female | SRCC | NHW | ≥55 | Male | 0.80 |

Abbreviations: NHW: Non-Hispanic White; NHB: Non-Hispanic Black, GIST: Gastrointestinal stromal tumor SRCC: Signet ring cell carcinoma.
