# Supplementary material for: Incidence trends of gastric cancer in the United States over 2000–2020: A population-based analysis
Source: PLoS One. 2024 Sep 25;19(9):e0310040. doi: 10.1371/journal.pone.0310040 (PMC11423999; doi:10.1371/journal.pone.0310040)
Supplement: S2 Table — (DOCX) [file pone.0310040.s002.docx]

**S2 Table.** Identical trends of gastric cancer incidence rate over 2000-2019 in the United States.

| Type | Race | Age | sex | Type | Race | Age | sex | P value |
| --- | --- | --- | --- | --- | --- | --- | --- | --- |
| Cohort 1 | | | | Cohort 2 | | | |  |
| Adenocarcinoma | Hispanic | <55 | Female | SRCC | Hispanic | <55 | Female | 0.18 |
| Carcinoid tumor | NHW | ≥55 | Female | GIST | NHW | ≥55 | Female | 0.20 |
| Gastric cancer | Hispanic | ≥55 | Female | Gastric cancer | NHB | ≥55 | Female | 0.67 |
| Adenocarcinoma | Hispanic | All | Female | Adenocarcinoma | NHB | All | Female | 0.18 |
| Adenocarcinoma | Hispanic | ≥55 | Female | Adenocarcinoma | NHB | ≥55 | Female | 0.27 |
| Carcinoid tumor | Hispanic | All | Male | Carcinoid tumor | NHB | All | Male | 0.88 |
| Carcinoid tumor | Hispanic | <55 | Male | Carcinoid tumor | NHB | <55 | Male | 0.37 |
| Carcinoid tumor | Hispanic | ≥55 | Male | Carcinoid tumor | NHB | ≥55 | Male | 0.46 |
| GIST | Hispanic | <55 | Both | GIST | NHW | <55 | Both | 0.53 |
| GIST | Hispanic | <55 | Female | GIST | NHW | <55 | Female | 0.42 |
| GIST | Hispanic | <55 | Male | GIST | NHW | <55 | Male | 0.38 |
| GIST | Hispanic | ≥55 | Male | GIST | NHW | ≥55 | Male | 0.41 |
| Carcinoid Tumor | NHB | ≥55 | Both | Carcinoid Tumor | NHB | ≥55 | Female | 0.19 |
| Carcinoid Tumor | NHB | ≥55 | Both | Carcinoid Tumor | NHB | ≥55 | Male | 0.25 |
| Carcinoid Tumor | NHB | ≥55 | Female | Carcinoid Tumor | NHB | ≥55 | Male | 0.38 |
| GIST | All | <55 | Both | GIST | All | <55 | Male | 0.06 |
| GIST | Hispanic | All | Both | GIST | Hispanic | All | Female | 0.13 |
| GIST | Hispanic | All | Female | GIST | Hispanic | All | Male | 0.10 |
| GIST | Hispanic | <55 | Both | GIST | Hispanic | <55 | Female | 0.30 |
| GIST | Hispanic | <55 | Both | GIST | Hispanic | <55 | Male | 0.36 |
| GIST | Hispanic | <55 | Female | GIST | Hispanic | <55 | Male | 0.37 |
| GIST | NHB | All | Both | GIST | NHB | All | Female | 0.22 |
| GIST | NHB | <55 | Both | GIST | NHB | <55 | Female | 0.45 |
| GIST | NHB | <55 | both | GIST | NHB | <55 | Male | 0.72 |
| GIST | NHB | <55 | Female | GIST | NHB | <55 | Male | 0.33 |
| GIST | NHB | ≥55 | Both | GIST | NHB | ≥55 | Female | 0.13 |
| GIST | NHW | <55 | Both | GIST | NHW | <55 | Female | 0.06 |
| SRCC | All | <55 | Both | SRCC | All | <55 | Female | 0.10 |
| SRCC | All | <55 | Both | SRCC | All | <55 | Male | 0.10 |
| SRCC | Hispanic | <55 | Both | SRCC | Hispanic | <55 | Female | 0.08 |
| SRCC | NHB | <55 | Both | SRCC | NHB | <55 | Female | 0.07 |
| SRCC | NHB | <55 | Both | SRCC | NHB | <55 | Male | 0.07 |

Abbreviations: NHW: Non-Hispanic White; NHB: Non-Hispanic Black, GIST: Gastrointestinal Stromal Tumor SRCC: Signet Ring Cell carcinoma
