# Supplementary material for: Incidence trends of gastric cancer in the United States over 2000–2020: A population-based analysis
Source: PLoS One. 2024 Sep 25;19(9):e0310040. doi: 10.1371/journal.pone.0310040 (PMC11423999; doi:10.1371/journal.pone.0310040)
Supplement: S4 Fig — Shaded areas are the confidence interval range for the point estimates. A: all race/ethnicity; B: Hispanics; C: Non-Hispanic Blacks; D: Non-Hispanic Whites. Note: Estimates were only provided for those with more than 16 cases. (DOCX) [file pone.0310040.s006.docx]

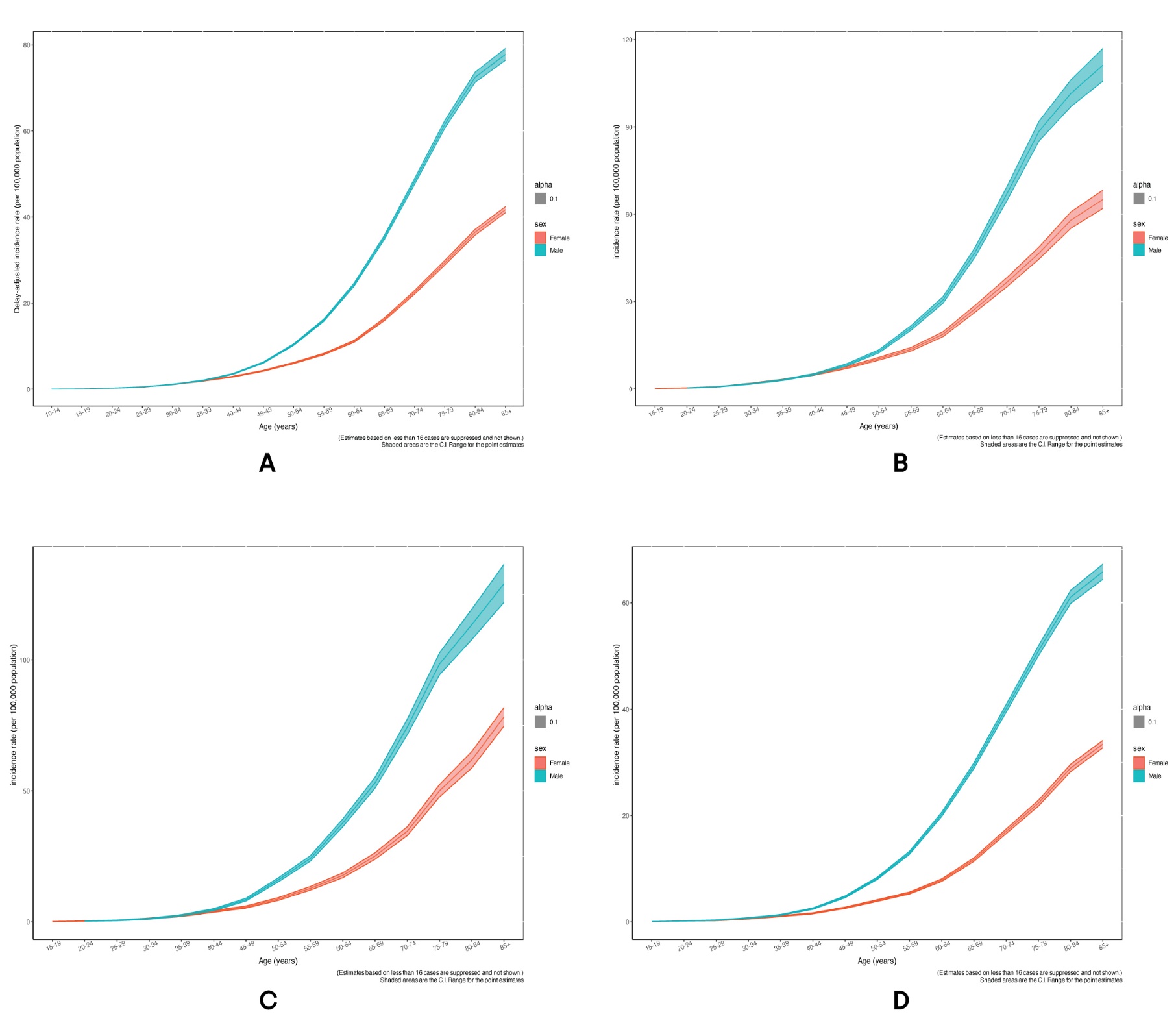


**S4 Fig.** Delay-adjusted incidence rate of gastric cancer in the United States among males and females in each age group. Shaded areas are the confidence interval range for the point estimates. A: all race/ethnicity; B: Hispanics; C: Non-Hispanic Blacks; D: Non-Hispanic Whites. Note: Estimates were only provided for those with more than 16 cases.
