# Supplementary material for: Incidence trends of gastric cancer in the United States over 2000–2020: A population-based analysis
Source: PLoS One. 2024 Sep 25;19(9):e0310040. doi: 10.1371/journal.pone.0310040 (PMC11423999; doi:10.1371/journal.pone.0310040)
Supplement: S8 Fig — APC: annual percent change. * Represent p-value less than 0.05. (DOCX) [file pone.0310040.s010.docx]

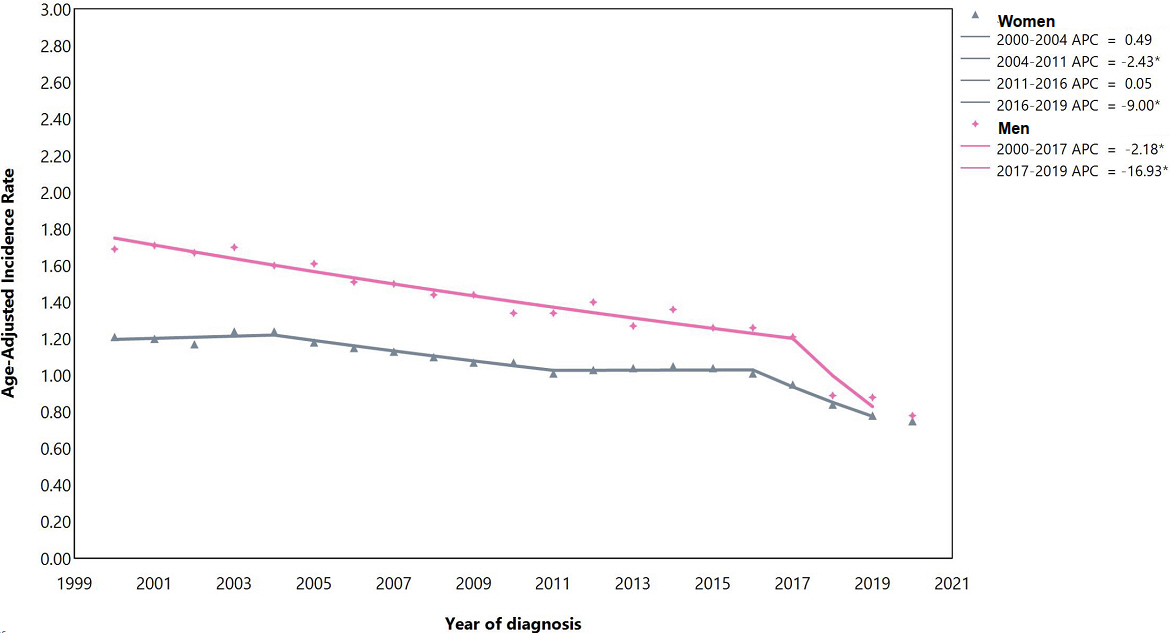


**S8 Fig.** Delayed age-adjusted incidence rate of signet ring carcinoma per 100,000 people over 2000-2019 and in 2020 in the United States, by sex. APC: annual percent change. * Represent p-value less than 0.05.
